# A Nationwide Cohort Study of Mortality Risk and Long-Term Prognosis in Infective Endocarditis in Sweden

**DOI:** 10.1371/journal.pone.0067519

**Published:** 2013-07-08

**Authors:** Anders Ternhag, Agneta Cederström, Anna Törner, Katarina Westling

**Affiliations:** 1 Department of Medicine, Solna, Division of Infectious Diseases, Karolinska Institutet, Stockholm, Sweden; 2 Department of Medical Epidemiology and Biostatistics, Karolinska Institutet, Stockholm, Sweden; 3 Department of Medicine, Huddinge, Division of Infectious Diseases, Karolinska Institutet, Stockholm, Sweden; National Institute of Allergy and Infectious Diseases, United States of America

## Abstract

*Objectives:* Infective endocarditis (IE) remains a serious disease with substantial mortality. In this study we investigated the incidence of IE, as well as its associated short and long term mortality rates.

**Methods:**

The IE cases were identified in the Swedish national inpatient register using ICD-10 codes, and then linked to the population register in order to identify deaths in the cohort. Crude mortality rates among IE patients were obtained for different time intervals. These rates were directly standardized using sex- and age-matched mortality in the general population.

**Results:**

The cohort consisted of 7603 individuals and 7817 episodes of IE during 1997–2007. The 30 days all-cause crude mortality rate was 10.4% and the standardized mortality ratio (SMR) was 33.7 (95% confidence interval [CI]: 31.0–36.6). Excluding the first year of follow-up, the long term mortality (1–5 years) showed an increased SMR of 2.2 (95% CI: 2.0–2.3) compared to the general population. Significantly higher SMR was found for cases of IE younger than 65 years of age with a 1–5 year SMR of 6.3, and intravenous drug-users with a SMR of 19.1. Native valve IE cases, in which surgery was performed had lower crude mortality rates and Mantel-Haenzel odds ratios of less than one compared to those with medical therapy alone during 30-day and 5-years follow-up.

**Conclusions:**

The 30-days crude mortality rate for IE was 10.4% and long-term relative mortality risk remains increased even up to 5 years of follow-up, therefore a close monitoring of these patients would be of value.

## Introduction

Infective endocarditis (IE) is a serious disease which is caused by a bacterial or fungal infection of endocardial surfaces of the heart, most often the valves. A lesion, or vegetation, which is composed by bacteria, platelets, leucocytes and fibrin can be visualized by echocardiography [1]. The age standardized incidence has been reported to be 2–7 cases per 100000 person years in different countries [2], [3]. The incidence in a previous 5-year period prospective study performed in Gothenburg, Sweden (using modified von Reyn criteria) was calculated to be 5.9 per 100000 and year [4]. Although the diagnostic criteria for endocarditis have changed [5] and transesophageal echocardiography has become more common, there are reasons to believe that the same incidence rate is true for other years and parts of Sweden. The short term mortality i.e. within 30 days from the diagnosis of disease is reported to be 10 to 30% in studies from different countries and depends on age, co-morbidity, etiology and whether it is a native or prosthetic valve IE [6]. There are also indications that the long-term mortality risk following an episode of IE is heightened. This could be due to recurrence of infection or complications of IE such as heart failure. However, it could also be a result of the changing patient groups of IE with underlying diseases and/or intravascular devices (as opposed to young adults with rheumatic valve disease or congenital heart abnormalities) [7], and an increase in incidence of Staphylococcus aureus IE [8].

In order to extend the body of research concerning the incidence of IE and its associated short- and long-term mortality risks, we conducted a large retrospective cohort study using record linkage. The aim of the study was to determine incidence, as well as short- and long-term crude mortality rates associated with IE among different categories of IE patients. We also calculated standardized mortality ratios for each of the categories.

## Materials and Methods

This survey was a nation-wide population-based register study of patients with IE. Our cohort consisted of patients who have been hospitalized and treated for IE during 1997 to 2007 in Sweden. The Swedish Hospital Discharge Register collects individual data from all hospitals and more than 99% of the discharges in somatic care are covered by the register [9]. In this study exposure was defined as a diagnosis of infective endocarditis (IE), and outcome was death or non-death by the end of the follow-up period. Exposure data (IE diagnosis) was collected from the nationwide inpatient register and outcome was then cross-referenced from the Swedish population register. Cases of IE were identified using ICD-10 codes: I33, I38, and I39. In order to refine the resulting mortality rates the cohort has been divided in several categories: native-valve IE, prosthetic-valve IE, intravenous drug users (IVDUs); as well as two age-groups; <65 years and ≥65 years. It can readily be assumed that different patient categories have different mortality risks, and therefore we wanted to isolate the rates specific to a particular patient profile. In addition to exposure (IE diagnosis) and outcome data, we also collected information on whether valve surgery was performed, or not performed, at any time point during follow-up up after initial hospital admission. The surgical operations of interest were identified using ICD-10 codes: FG, FJ, FK, and FM.

For the patients in our cohort we have established the date, but not specific cause, of death (if it occurred before the end of our follow-up period). Therefore it was the all-cause attributable IE mortality rates which were determined. Crude rates were determined by counting the number of deaths in our patient cohort within a specific time period after IE was diagnosed. The crude mortality rates represented the absolute mortality risk in the cohort. Another way to illustrate the absolute mortality risk associated with IE is to look at the Kaplan-Meier survival estimator, where the crude survival rate is 1- crude mortality rate.

In order to explore possible increases in long-term relative mortality risks, the crude mortality rates were then directly standardized using age- and sex- stratified mortality rates from the general population of Sweden as the reference population. This data was available from the Statistics Sweden. The standardized mortality ratio (SMR), was then the ratio between the observed number of deaths and the expected number of deaths, where the expected number of deaths was obtained by multiplying the person-years in the cohort with the age- and sex specific mortality rates in the Swedish general population. Ninety-five percent confidence intervals for the SMR were calculated assuming that the observed number of deaths was Poisson distributed. Comparisons of mortality between early surgery and medical therapy were done within each patient category (native and prosthetic valve IE) using age- and sex- stratified Mantel-Haenszel estimates of the odds ratio. The time trend for the annual incidence and mortality rate of IE was explored in a linear regression model using a quasipoisson distribution and t-test for significance.

### Ethics Statement

This study was approved by the research ethics committee at Karolinska Institutet, Stockholm. The National Board of Health and Welfare insured patient confidentiality by replacing all personal identity numbers with serial numbers in the data set.

## Results

**Figure 1:**
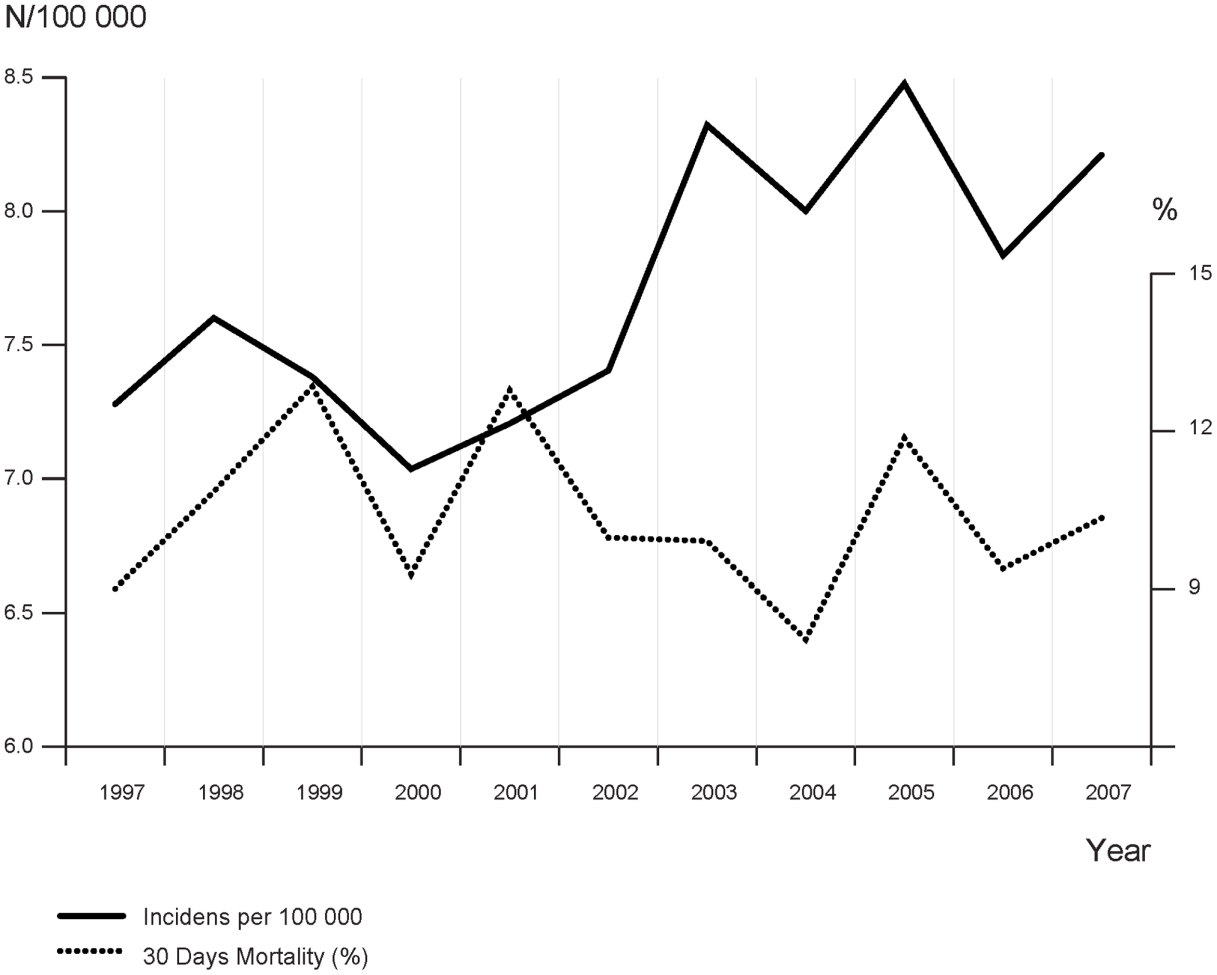
Incidence Rate and 30-days Mortality (%) of Infective endocarditis (IE) hospitalizations in Sweden during 1997 through 2007.

In Sweden between 1997 and 2007, 7603 individuals were diagnosed with 7817 cases of IE. This corresponds to an average annual incidence of 7.7 per 100000. The incidence rate has increased somewhat during the study period (slope of the line 0.01, p-value for trend 0.01) (Figure 1). There were more males (59.2%) than females (40.8%) in the cohort and the mean age was 65.7 years (interquartile range [IQR]: 55–79 years). The median time for hospital stay was 23 days (IQR: 9–37 days).

Of the 7603 patients in the cohort, about 80% had native valve IE, 12% prosthetic valve IE, and 5% were intravenous drug users. A few persons had both a current intravenous drug use and a prosthetic valve and they are categorized among drug-users in the analysis. The IVDUs were a young subpopulation in the cohort, with a mean age of 39 years (IQR: 31–45 years).

**Figure 2:**
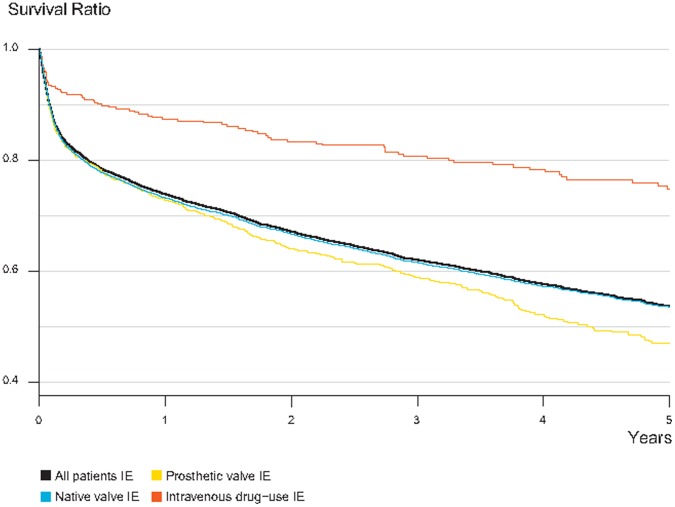
Absolute Mortality Risks in the Total Infective endocarditis (IE) Cohort (n = 7603) and grouped by Native Valve IE (n = 6138), Prosthetic Valve IE (n = 890) and Intravenous drug-users (n = 575), 5-year Follow-up. Individuals who both have a current intravenous drug use and a prosthetic valve are categorized among drug-users.

For all patients in the cohort, the all-cause 30-days crude mortality rate was 10.4% (Table 1). The mortality rate fluctuates annually during 1997–2007 with no obvious trend through the years (slope of the line −0.006, p-value for trend 0.7) (Figure 1). The Kaplan-Meier survival estimator for the cohort showed a sharp decline in survival during the first few months and then gradually leveling of absolute mortality risk through the 5-year period (Figure 2). Only 23 of 355 of intravenous drug users died in the first 30 days, corresponding to a crude mortality rate of 6.5%, compared to 10.5% and 11.2% for patients with native valve and prosthetic valve IE, respectively. No significant differences in absolute or relative mortality risks were found between patients with native valve and prosthetic valve IE during 1-year follow-up, but those with prosthetic valve IE had a lower 5-year survival.

**Table 1:** Demographic data and All Cause 30-days Crude Mortality Rates (%) among Different Categories of Infective endocarditis (IE) Subjects (n = 7603).

Subjects	No. of patients, (% men)	Age, mean (IQR)	Crude 30-days Mortality, (%)
**Total**	7609 (59.2)	65.7 (55–79)	788 (10.4)
**Categories**			
Native-valve	6138 (57.6)	66.8 (57–80)	642 (10.5)
Prosthetic-valve	890 (62.7)	70.4 (65–79)	100 (11.2)
Intravenous drug users	355 (64.2)	38.8 (31–45)	23 (6.5)
**Age**			
≤65 years	3074 (67.5)	N/A	208 (6.8)
>65 years	4529 (53.5)	N/A	580 (12.8)
**Gender**			
Men	4498	63.6 (53.0–77.0)	447 (9.9)
Women	3105	68.7 (59.0–82.0)	341 (11.0)
**Surgery**			
Total Surgery	990 (73.7)	55.5 (44–67)	62 (6.3)
Total Non-Surgery	6613 (57)	67.2 (58–80)	726 (11)
Native-Valve Surgery	778 (72.1)	55.8 (47–67.8)	42 (5.4)
Native-Valve Non-Surgery	5360 (55.5)	68.4 (60–81)	600 (11.2)
Prosthetic-Valve Surgery	104 (74)	61.3 (56.8–72)	16 (15.4)
Prosthetic-Valve Non-Surgery	786 (61.2)	71.6 (67–80)	84 (10.7)

Individual who both have a current drug use and a prosthetic valve are categorized among drug-users.

IQR, Interquartile range.

The standardized 30-days mortality ratio (SMR) was 33.7 (95% confidence interval [CI]: 31.0–36.6) for all patients in the cohort. The SMR decline with time, and was 3.9 (95% CI: 3.6–4.3) for the period of 3 to 12 months after the IE diagnosis. It remains greater than one even after one year, where the 1 to 5 year SMR was 2.2 (95% CI: 2.0–2.3).

This long term relative mortality risk during 1 to 5 year follow-up varied between the categories within the cohort and was highest for patients ≤65 years of age, SMR 6.3 (95% CI: 5.5–7.2) and for IVDUs, SMR 19.1 (95% CI: 13.5–27.1) (Table 2).

**Table 2:** Long-term Mortality in the Infective endocarditis Cohort compared to the Age- and Sex-matched Swedish General Population (n = 7603).

	Time 1–5 Years			
Subjects	Obs. No. of Deaths, (%)	Exp. No. of Deaths	SMR	95% CI
**Total**	1117 (14.7)	518.6	2.2	2.0–2.3
**Categories**				
Native-valve	894 (14.6)	441.9	2.0	1.9–2.2
Native-valve Men	492 (13.9)	247.6	2.0	1.8–2.2
Native-valve Women	402 (15.5)	194.4	2.1	1.9–2.3
Prosthetic-valve	154 (17.3)	67.9	2.3	1.9–2.7
Prosthetic-valve Men	79 (14.2)	41.0	1.9	1.5–2.4
Prosthetic-valve Women	75 (22.6)	26.9	2.8	2.2–3.5
Intravenous drug users	32 (9.0)	1.7	19.1	13.5–27.1
**Age**				
≤65 years	228 (7.4)	36.3	6.3	5.5–7.2
>65 years	889 (19.6)	482.3	1.8	1.7–2.0
**Gender**				
Men	623 (13.9)	296.1	2.1	1.9–2.3
Women	494 (15.9)	222.5	2.2	2.0–2.5

IQR, Interquartile range; Obs, Observed; Exp, Expected; SMR, Standardized mortality ratio; CI, Confidence interval.

**Figure 3:**
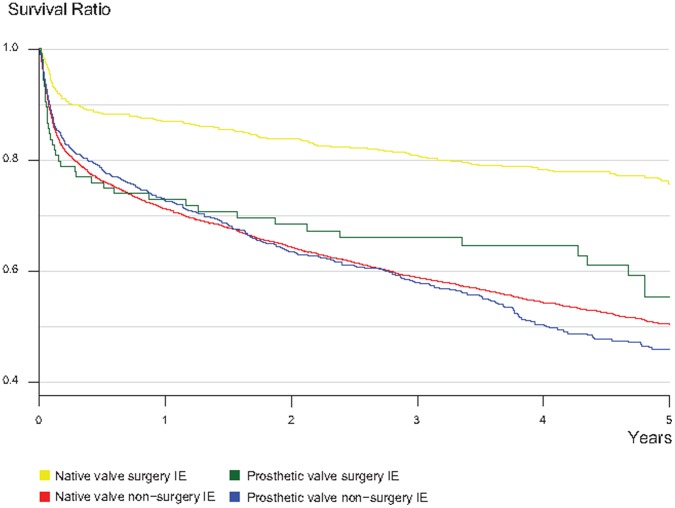
Absolute Mortality Risks among Infective endocarditis (IE) Patients grouped by Native Valve IE surgery (n = 881), Native Valve IE non-surgery (n = 5257), Prosthetic Valve IE surgery (n = 109) and Prosthetic valve IE non-surgery (n = 781), 5-year Follow-up.

We also calculated crude mortality rates among patients in the cohort who underwent surgery for native and prosthetic valve IE and compared these rates to those patients who did not undergo surgery. Of the 7603 patients, 990 underwent surgery, which was approximately 13% of the cohort (of the 990 surgical patients, 881 had native valve IE and 109 had prosthetic valve IE). Those patients who underwent surgery tended to be younger with a mean age of 55.5 years (IQR: 46–67 years) and had a higher proportion of male patients 73.7%, compared to those who did not have surgery which had a mean age of 67.2 years (IQR: 58–80) and 58% male. Patients with surgery had a lower crude mortality rate compared to those who did not undergo surgery during a 1-year follow up. However, there was a marked difference in absolute mortality risks for between patients with native valve and prosthetic valve IE among those who underwent surgery (Figure 3). The crude 30-days mortality rate for those with native valve IE, who underwent surgery was 5.2% (46/881) compared to 11.0% (640/5812) in cases where surgery was not performed. The reverse was true for prosthetic valve IE, where patients who underwent surgery had a crude 30-days mortality rate of 14.7% (16/109) compared to 10.7% (86/801) for those who did not undergo surgery. After 5-years follow-up, survival was highest among those with surgery irrespective of native or prosthetic valve IE.

To correct for the age- and sex effects we stratified the groups, and then calculated Mantel-Haenszel adjusted mortality odds ratios (OR) for surgery to non-surgery for IE patients in the different categories. The Mantel-Haenszel 30-days mortality odds ratio (surgery to non-surgery) for all the patients in the cohort was 0.7 (95% CI: 0.5–1.0), which indicates that those patients who underwent surgery had better chances of survival. When distinguishing between native valve and prosthetic valve IE we found the same effect where native-valve IE had a 30-day mortality OR of 0.6 (95% CI: 0.4–0.9) comparing surgery to non-surgery while the group with prosthetic-valve IE had an OR of 2.2 (95% CI: 1.1–4.4). Similar results were found when the follow up period was lengthened to 1 year (Table 3).

**Table 3:** Comparison of Mortality Risks between Patients with Infective endocarditis treated with Medical Therapy Alone (n = 6613) and Valve Surgery (n = 990) using Mantel-Haenszel Statistics.

	Time 0–1 months		Time 0–12 months	
Subjects	Mortality ratio	95% CI	Mortality ratio	95% CI
All subjects (Surgery/No surgery)	0.7	0.5–1.0	0.7	0.6–0.9
Native-valve (Surgery/No Surgery	0.6	0.4–0.9	0.7	0.5–0.9
Prosthetic-valve (Surgery/No Surgery)	2.2	1.1–4.4	1.7	0.9–3.1

## Discussion

This large nation-wide retrospective register-based cohort study provided precise estimates of the all-cause mortality risk associated with infective endocarditis (IE) for a five year follow up period, the vast majority of bacterial etiology. The incidence of IE in the present study, 7.7 per 100000 persons per year, was higher than in a previous population-based study from Gothenburg, Sweden, during 1984–1988, including 99 prospective episodes of IE, and 34 episodes included retrospectively [4]. Clinical, microbial and autopsy data were available for each patient. A lower incidence of IE has been reported in repeated population-based prospective studies from France [10], the latest performed in 2008 [11] including 497 patients, with an incidence of 3.4 per 100000 person-years, and clinical data were available for the patients. A study from US during 1999–2008, had a higher and increasing incidence of IE, 16 per 100000 person-years in 2006 [8]. That study performed retrospectively on a nationwide in-patient sample database, by using ICD-9 codes, including 83700 patients. Etiological agents were analyzed due to ICD classification codes, but other clinical data were not available.

Our main finding was an all cause 30 days crude mortality rate of 10.4% among 7603 persons with IE which corresponded to a standardized mortality ratio (SMR) of 33.7. An increase in relative mortality risk was evident a long time after the infection with a 1 to 5 year SMR of 2.2, where those younger than 65 years and IVDUs had even higher relative risk ratios.

Our finding that SMR was highest at the initial phase of IE and gradually diminishes with time, but still not equal to the general population 5 years after the acute disease has been supported by other studies [12]–[14]. The in-hospital mortality contributed to most of the observed deaths. However, even after excluding in-hospital and first year mortality, there was still an increased mortality risk compared to the general population. There could be several explanations to this finding. One reason is that some IE cases engender complications (i.e. heart failure) [15] which increase mortality risk; another is that already vulnerable individuals with underlying diseases and intravascular devices are at a greater than average risk of acquiring IE and their base-line mortality risk is higher than the general population.

Intravenous drug use is an established risk factor for IE. While IVDU patients were younger and had more right-sided IE [16], which could explain their relatively low observed absolute 30-day mortality risk compared to the other patient groups in our study, their long-term standardized mortality ratio (SMR) was as high as 19.2, likely due to the low expected mortality in the corresponding age groups in the general population.

There was probably a strong selection bias that explains the diverging results concerning mortality after surgery among native-valve and prosthetic-valve IE, as the former had a lower mortality odds ratio within 30 days with surgery compared to non-surgery, and the latter had a higher mortality odds ratio. In both groups the younger and probably those with no co-morbidities probably were more likely to have surgery than the oldest and most vulnerable individuals, as earlier has been described [17]. In an observational study on prosthetic valve IE and mortality, these selection problems were illustrated by stratifying patients into three groups: surgery, deliberately conservative and perforced conservative medical treatment [18]. The lowest mortality risk was shown for deliberately conservative, followed by surgery, and highest among perforced conservative.

Taken all together, 6.3% of those who had surgery died during the first 30 days compared to 11% of those who did not, which was an in-hospital risk reduction attributable to surgery found at other places [19]. Among those with prosthetic valve IE, those with late infection (>12 months after implantation), non *S. aureus* etiology and no perivalvular spread of infection are more likely to respond to antibiotics alone [1], whereas more complicated cases (with subsequent higher mortality risk) need surgery. However, the findings in our study of a lower absolute mortality risk at every time point during follow-up among those with native IE who had valve surgery, is supported in other retrospective cohort studies where efforts have been made to handle biases in treatment assignment and underlying morbidity risks [20], [21]. The proportion of cases, in which surgery was performed was 13% in our data, and this could be an underestimation. We identified surgery due to IE by combining ICD-codes for IE and valve surgery at discharge, but if any of these codes for surgery were missing (although surgery was performed), we would miss counting surgery for that specific patient.

Our study has some draw-backs: in particular, detailed clinical and microbiological information was not available, which limits this study considerably. It would have been of interest to have information about comorbidity because it affects the survival of endocarditis [14], [22] and is also a factor to be taken into account whether surgical treatment is carried out or not [23]. Different bacterial etiologies have different prognosis in IE; in particular, it is well known that left-sided S. *aureus* IE is associates with poorer survival compared with viridans group streptococci [11], [24], [25]. The Swedish Hospital Discharge Register has great coverage, and therefore a high sensitivity in identifying all cases of IE, but the specificity for IE discharge diagnoses in the register has not been externally validated. Almost all patients with IE in Sweden are hospitalized. Outpatient parenteral antibiotic treatment (OPAT) is mainly performed in patients with uncomplicated IE caused by viridans group streptococci, in which antibiotic treatment is initiated at hospital, and continued in outpatient care or advanced home health care. The use of record linkage between our national databases means that in practice only a negligible proportion of patients are lost to follow-up (solely those who have emigrated during study period).

This Swedish nationwide cohort study of IE cases estimates the 30-days absolute mortality risk to be about 10%, which is low. Excluding the first year of follow-up, the long-term relative mortality risk was 2.2 times higher in the patient cohort compared to the general population, which suggests that a follow-up of these patients would be of value.
